# Are role perceptions of residents and nurses translated into action?

**DOI:** 10.1186/s12909-017-0976-2

**Published:** 2017-08-18

**Authors:** Naïke Bochatay, Virginie Muller-Juge, Fabienne Scherer, Guillemette Cottin, Stéphane Cullati, Katherine S Blondon, Patricia Hudelson, Fabienne Maître, Nu V Vu, Georges L Savoldelli, Mathieu R Nendaz

**Affiliations:** 1 Unit of Development and Research in Medical Education (UDREM), Faculty of Medicine, University of Geneva, Rue Michel Servet 1, 1211 Geneva, Switzerland; 2 Geneva University Hospitals, Geneva, Switzerland; 3 Faculty of Medicine, University of Geneva, Geneva, Switzerland; 4 Quality of Care Service, Geneva University Hospitals, Geneva, Switzerland; 5 Division of General Internal Medicine, Geneva University Hospitals, Geneva, Switzerland; 6 Department of Community Medicine, Primary Care and Emergency Medicine, Geneva University Hospitals, Geneva, Switzerland; 7 Division of Anesthesiology, Geneva University Hospitals, Geneva, Switzerland

**Keywords:** Interprofessional collaboration, Interprofessional education, Professional identity, Role perception, Role clarity, Mixed methods

## Abstract

**Background:**

Effective interprofessional collaboration (IPC) has been shown to depend on clear role definitions, yet there are important gaps with regard to role clarity in the IPC literature. The goal of this study was to evaluate whether there was a relationship between internal medicine residents’ and nurses’ role perceptions and their actual actions in practice, and to identify areas that would benefit from more specific interprofessional education.

**Methods:**

Fourteen residents and 14 nurses working in internal medicine were interviewed about their role perceptions, and then randomly paired to manage two simulated clinical cases. The authors adopted a general inductive approach to analyze the interviews. They identified 13 different role components that were then compared to data from simulations. Descriptive and kappa statistics were used to assess whether there was a relationship between role components identified in interviews and those performed in simulations. Results from these analyses guided a further qualitative evaluation of the relationship between role perceptions and actions.

**Results:**

Across all 13 role components, there was an overall statistically significant, although modest, relationship between role perceptions and actions. In spite of this relationship, discrepancies were observed between role components mentioned in interviews and actions performed in simulations. Some were more frequently performed than mentioned (e.g. “Having common goals”) while others were mentioned but performed only weakly (e.g. “Providing feedback”).

**Conclusions:**

Role components for which perceptions do not match actions point to role ambiguities that need to be addressed in interprofessional education. These results suggest that educators need to raise residents’ and nurses’ awareness of the flexibility required to work in the clinical setting with regard to role boundaries.

## Background

In the field of healthcare, interprofessional collaboration (IPC), which refers to health professionals from different occupational groups working together to care for patients [1], has become increasingly important in the past decades [2]. Analyzing how healthcare teams work together, Grumbach and Bodenheimer [3] observed that having common and well-defined goals, institutional stability, being trained to perform one’s role, communication, and role clarity were essential elements of IPC. Professional role therefore represents an important aspect of IPC [4].

As the medical education literature often overlooks clarifying what is meant by professional role [5], we adopted Heiss’ definition and considered that roles represent “a set of expectations in the sense that it is what one *should* do” [6]. Although we are aware of the importance of structural factors in shaping healthcare professionals’ roles [5], our interpretation of roles is more in line with the symbolic interactionism tradition [7], which views them as being constructed and negotiated through social interactions [8]. This understanding of professional roles allows distinguishing between how roles are perceived on the one hand, and individuals’ actual role performances on the other hand. It also suggests that discrepancies between role expectations and role performance may be identified [9], a phenomenon that needs to be better understood in the context of interprofessional collaboration.

Historical perspectives on the evolution of professions and local practice regulations may influence healthcare professionals’ actions [10]. In spite of the recognition of the nursing profession at the beginning of the twentieth century, the culture of some medical institutions may still be characterized by historical tradition and influence role perceptions (e.g., “doctor-nurse game”) [11, 12] and social hierarchies between healthcare professions [13]. Depending on the country, political, economical, and professional regulations determine the range of practice of physicians and nurses, thus influencing their actions. However, individual factors may also affect the link between perceptions and actions. For example, Allen’s work uncovered differences between what British nurses aspired to and the actual work they performed [14, 15]. Similar observations were made by McGarvey, Chambers, and Boore [16]. In an exploratory research on operating room nursing combining interviews and observations, they described how nursing was both viewed and performed. There were significant differences between the two that impacted both the work of healthcare teams and patient care: while nurses valued their relationship with patients in interviews, they had little interactions with them in practice, preferring to assist physicians and to work with technology than to provide emotional support to patients [16].

Further work on role clarity in healthcare has stressed the limited knowledge that professionals have of groups other than their own, and the negative impact this could have on interprofessional collaboration [4, 17, 18]. Van Schaik and colleagues [19] identified knowledge of one’s own role, as well as of others’ roles, as critical to IPC in lower-acuity settings. Such settings need particular attention when conducting research on interprofessional collaboration, as they are usually characterized by structures that are becoming more collaborative and less rigid [19, 20], and as these settings require healthcare professionals to communicate in an efficient way to deal with increasingly complex cases [21]. Internal medicine represents one example of such lower-acuity settings. While several studies have looked at IPC in internal medicine, including a large, multi-site project that focused on interprofessional communication in healthcare teams [22], they have not addressed the issue of role clarity in relation to IPC specifically.

The goal of this study was to evaluate whether there was a relationship between internal medicine healthcare professionals’ role perceptions and their actions in practice. Considering that residents and nurses represent the two occupational groups that are the most involved in first-line patient care in internal medicine in the Swiss context, our study focused on these two groups. Furthermore, we sought to identify potential gaps to inform interprofessional training programs.

## Methods

### Design

The work presented here is part of a larger project on IPC. This project involved two main steps: first, individual interviews were conducted with 14 residents and 14 nurses in internal medicine about their perceptions and expectations regarding both their own and the other profession’s roles. Two weeks later, 14 resident-nurse pairs were randomly created with the same participants and each pair managed two clinical cases in a simulated ward, using a high-fidelity manikin. This second step allowed for the analysis of behaviors enhancing the quality of patient management. The methodology for each step is detailed in previous publications [10, 17], but a summary is provided below.

For this paper, we identified components that participants associated with their professional role in the individual interviews and compared them with actions performed in simulations, so as to understand whether there was a relationship between the two.

### Setting and participants

This research project was conducted in the in-patient general internal medicine division of a Swiss 1800-bed teaching hospital. Eligibility criteria included having between one and 5 years of experience in internal medicine for residents, and working on the internal medicine ward for nurses; substitute residents and nurses were excluded. At the time of data collection, a total of 33 residents and 54 nurses were eligible. Potential candidates were told about the research during staff meetings, and could thereafter volunteer to take part in it. Participants were recruited throughout the first step of the research, until saturation was reached in the interview data [23, 24]. In total, we recruited 14 residents and 14 nurses. Participant characteristics are shown in Table 1.

**Table 1:** Participant Characteristics

	Residents	Nurses	Total
N	14	14	28
Gender (N male:N female)	10:4	4:10	14:14
Mean age (range)	31 (25; 36)	37 (27; 48)	34 (25; 48)
Mean years of experience (range)	4 (0.5; 7)	10 (2; 25)	7 (0.5; 25)
Mean years of experience in the Division of General Internal Medicine (range)	3 (0.5; 5)	4 (0.5; 13)	3 (0.5; 13)

### Data collection

The first step of the research [17] aimed at exploring residents’ and nurses’ role perceptions through individual, semi-structured interviews. All interviews were audio-taped and conducted by an educationalist who had been trained in qualitative research methods (VMJ), using an interview guide that had previously been tested with residents and nurses who did not participate in the study. At the end of each interview, clinical vignettes of cases commonly encountered in internal medicine were used to evaluate role perceptions in a more contextualized way. In the second step of the research [10], each resident was randomly paired with a nurse, and all 14 resident-nurse pairs managed two clinical cases in a simulated environment. Similar to the vignettes that had been used in interviews, these cases represented typical situations of ward rounds or urgent on-call events. These simulations were videotaped, and followed by individual stimulated-recall sessions (VMJ, SC, NVV, GLS, MRN) during which participants watched the videos of their simulations and commented on their actions, either spontaneously or when prompted by researchers [25].

Verbal utterances of interviews, simulations, and stimulated-recall sessions were transcribed verbatim; actions in simulations were transcribed and double-checked by members of the research team (VMJ, MRN, FM). The transcripts were coded systematically, using Atlas.ti (ATLAS.ti Scientific Software Development GmbH, Berlin, Germany, Version 6.2.18).

### Data analysis

#### Interviews

To identify key role components in our interview data, we adopted a general inductive approach [26]. We read and coded interview transcripts iteratively, until we agreed on a coding scheme that represented 13 components of participants’ professional roles. The 13 role components were classified as belonging to one of three main categories: 1) autonomy, reflection, and leadership, 2) technical communication, which notably refers to verifying the other’s work, sharing technical information, and planning the work sequence, and 3) team support (Table 2).

**Table 2:** Categories and role components

1. Autonomy, reflection, and leadership	
1.1	Making shared decisions
1.2	Having common goals
1.3	Being proactive and making decisions
1.4	Depending on the other
1.5^a^	Being involved in case understanding
1.6^a^	Making suggestions
2. Technical communication	
2.1	Sharing technical information
2.2	Verifying the other’s work
2.3^a^	Planning
3. Team support	
3.1	Providing feedback and support
3.2^b^	Providing training
3.3	Being available, providing help
3.4	Contributing to team building

^a^ *Role components mentioned by nurses only*

^b^ *Role components mentioned by residents only*

#### Simulations

The coding scheme developed with the interview data (Table 2) was used to code data from simulations and stimulated-recall sessions, according to a template analysis approach [27, 28]. This approach allowed assessing whether and how role components identified in interviews (perceptions) were translated into action, using participants’ role perceptions as a reference. Participants’ comments in the individual stimulated-recall sessions enabled us to better understand why they had performed specific actions. To avoid biasing our results, participants knew that we were interested in role perceptions and in collaborative practice, but they were not specifically aware of our intention to compare the two.

#### Relationship between interviews and simulations

**Fig. 1:**
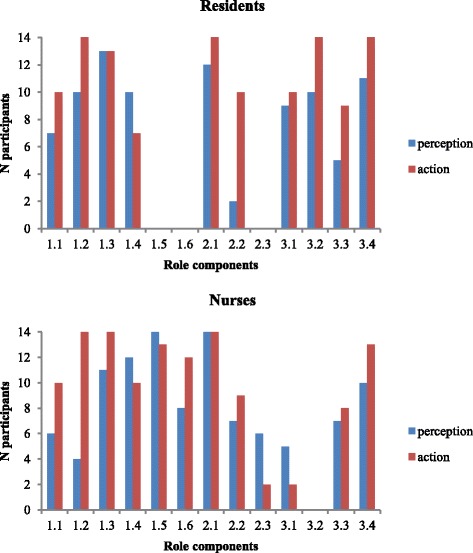
Number of participants having expressed each role component as perceptions in interviews and as actions in simulations

To assess the relationship between role components discussed in interviews and the actions we observed in simulations, we used an explanatory mixed methods design where quantitative results guided the subsequent qualitative analysis of our data [29]. Our design involved three stages. First, the data of both steps of the research were tabulated in a database organized by role component and by participant. Two independent researchers (VMJ, MRN) evaluated whether each of the 13 components listed in Table 2 was expressed both as a role perception during interviews and in action during simulations (inter-coder kappa for role perceptions: 0.87, *p* < 0.001; inter-coder kappa for actions: 0.76, *p* < 0.001). Disagreements were solved by consensus. Second, for our quantitative analysis, we evaluated the number of participants who mentioned role components during interviews and who performed them in simulations (Fig. 1). Overall concordance between role perceptions and actions was assessed using kappa statistics that were computed for residents and nurses separately, and for all participants (SPSS statistical software, SPSS Inc., Chicago, IL, USA, Version 23). Results from these analyses guided the third stage of our analysis. This stage represented a qualitative evaluation of data from our interviews, simulations, and stimulated-recall sessions to better understand the nature of the relationship between role perceptions and actions (NB, FS, GC). Results of this third stage were shared with the rest of the group for review comments and refinement on a regular basis.

## Results

### Concordance between role perceptions and actions

For all role components (193 total occurrences in interviews and 240 occurrences in simulations) and participants, there was an overall statistically significant, although modest, relationship between role perceptions and actions. Kappa coefficients were 0.20 for residents (*p* = 0.007), 0.27 for nurses (*p* < 0.001), and 0.25 for all participants (*p* < 0.001). Fig. 1 shows the number of participants who mentioned each role component in interviews and/or performed it in simulations, and allows a comparison between residents and nurses. Even though Fig. 1 sheds light on very concordant or discordant role components, it fails to represent how strongly or how frequently role components were mentioned or performed. This analysis is also limited in its ability to inform us on the relationship between perceptions and actions. Therefore, we adopted a qualitative approach to better understand these initial quantitative results.

### Qualitative analysis

As shown in Fig. 1, nine role components were identified among both residents and nurses [17], including “Sharing technical information” and “Contributing to team building”. Additional role components were more strongly associated with one single group: “Providing training” to nurses was more strongly expressed by residents, while “Being involved in case understanding”, “Making suggestions”, and “Planning” was more strongly expressed by nurses. Table 3 summarizes our qualitative evaluation of the concordance strength of role components for residents and for nurses. In this section, we first present components for which role perceptions tended to concur with actions, before moving to components for which role perceptions did not match actions as strongly. Given the number of components identified in our data, we illustrate those with a clear pattern or those emphasized by participants, and present results for residents and nurses separately.

**Table 3:** Qualitative evaluation of the concordance strength of role components

Role Perceptions	Actions	
	Weak expression	Strong expression
**Residents**		
**Weak expression**		1.1 Making shared decisions 1.2 Having common goals 2.2 Verifying the other’s work 3.3 Being available, providing help
**Strong expression**	1.4 Depending on the other	1.3 Being proactive and making decisions 2.1 Sharing technical information 3.1 Providing feedback and support 3.2^b^ Providing training 3.4 Contributing to team building
**Nurses**		
**Weak expression**		1.1 Making shared decisions 1.2 Having common goals 1.6^a^ Making suggestions 2.2 Verifying the other’s work 3.3 Being available, providing help
**Strong expression**	1.4 Depending on the other 2.3^a^ Planning 3.1 Providing feedback and support	1.3 Being proactive and making decisions 1.5^a^ Being involved in case understanding 2.1 Sharing technical information 3.4 Contributing to team building

^a^ *Role components mentioned by nurses only*

^b^ *Role components mentioned by residents only*

### Concordant role components

#### Residents

Several components of residents’ roles were particularly concordant, including “Being proactive and making decisions”, “Sharing technical information”, and “Contributing to team building”, which refers to residents’ ability to respect and listen to nurses, and to create a positive working environment. Such endeavor was notably highlighted by a resident:

*I have always managed to build relationships based on trust with nurses I have worked with. I knew they would call me if they needed to, and they knew I would be available if they called me. (R5, interview)*

In simulation, this resident listened to N5’s recommendations and thanked her when she made suggestions. She also ensured that the nurse would call her if she left the room:

*R5: I’m just going to ask the fellow to come over, I don’t really want to slow the patient’s heart down…*

*N5: Sure, you can call him.*

*R5: Could you just stay with the patient while I’m on the phone?*

*N5: Of course.*

*R5: And just call me if there’s anything, I’ll be right there. Thanks!*

During stimulated-recall sessions, R5 said that she was very happy with her interaction with the nurse.

*It went really well, I could feel, I mean, it depends on the nurse, but N5 was on top of things, she didn’t get stressed or anything, and she was able to make suggestions. So that was good, because I may not think about everything if I’m focused on something else, so it’s good to have someone who is more focused on more concrete things, who asks the right questions: what are we going to do next? What can we do now? And in a constructive way. She made suggestions, I thought it was great. (R5, stimulated-recall session 2)*

#### Nurses

Nurses were concordant on role components such as “Being involved in case understanding”, which refers to nurses’ involvement in a given situation, and “Sharing technical information”, which was identified by nurses’ stressing the importance of communicating with residents, and by their sharing information with the latter. One nurse viewed this as a key element of IPC:

*During morning rounds, we tell physicians what happened the previous day, what needs to be changed, how patients are doing, whether they feel better or got worse. Collaboration starts there. (N13, interview)*

During simulations, this nurse asked R13 to come and see a patient. Whereas other nurses simply let residents talk to patients to evaluate them, N13 looked at R13 and told her why she had paged her as soon as she got in the room:

*This patient just called me because his abdomen hurts. He pointed at his stomach and said that the pain is constant. It doesn’t increase when I press on his stomach or when he breathes. I didn’t ask if it radiated… (N13, simulation 2)*

### Less concordant role components

#### Residents: Weaker role perceptions, stronger actions

Among less concordant components of residents’ role, “Verifying the other’s work” was more frequently performed in practice than it had been mentioned in interviews, as was the case for “Having common goals” (assessed by residents’ ensuring that nurses understood the situation). While few residents mentioned that sharing goals with nurses was part of their roles, their actions in simulations stressed this component. This could be done by directly communicating with nurses:

*Okay, gram-positive bacteremia, which means that we can start giving him antibiotics immediately, and I’ll just call my supervisor to let him know… (R7, simulation 2)*

Residents sometimes ensured that nurses knew what was happening by talking to them through patients:

*[To the patient] My colleague is going to take a blood sample to know what blood type you are. Then we will order some blood units. (R13, simulation 1)*

In her stimulated-recall session, R13 explained that she felt that she could communicate with N13 through the patient because they shared the same goals and had both understood the situation:

*I think she knows what’s going on, we are on the same page. I feel like I don’t necessarily need to say everything I think, she has understood, and we are going to transfuse the patient. N13 offered to do it herself, so it really shows that she knows what’s going on. (R13, stimulated-recall session 1)*

#### Nurses: Weaker role perceptions, stronger actions

Among nurses, “Having common goals” was also discordant: only a few nurses mentioned this role component in interviews, but all of them undertook actions that demonstrated their sharing objectives with residents. “Making suggestions”, which was specific to nurses, was also more strongly enacted than it was referred to in interviews.

*R2: Could you stay with the patient? I need to call the resuscitation team.*

*N2: Sure. Should I give him some oxygen?*

*R2: You can give him some, his blood saturation is good but he’s in shock.*

Similar to N2, most nurses made regular suggestions and anticipated residents’ needs in simulations, although they had not stressed is as being part of their role in interviews. Such mismatch may be explained by nurses’ lack of awareness of what they usually do when they work:

*I realize that we sometimes focus on things because we think that’s what it is, and we don’t pay attention to the rest. (N2, stimulated-recall session 2)*

#### Residents: Stronger role perceptions, weaker actions

Failing to translate what had been mentioned during interviews into action represented another group of discordant role components. The only component that was discordant in this way among residents was “Depending on the other”. It was assessed by residents’ claims and actions pertaining to role complementarity, their dependence on nurses, and their acknowledgement of role boundaries and of their own limits. In interviews, most residents said they depended on nurses:

*If, for a whole weekend, we removed all nurses from the hospital, there would be a skyrocketing number of deaths. If we removed doctors, there would be a lot less deaths […]. What I’m trying to say is that we have different roles, but we really are part of the same system. Without either profession, it wouldn’t work as well, but I feel that without nurses, it would work even less than without physicians. (R6, interview)*

In simulations, however, residents tended to act autonomously, without depending on nurses for their own actions. They usually examined patients and talked to them, and told nurses about the next steps without asking for their help or opinion:

*Okay… (stares at the screen, then talks to N6) Just before we give him bronchodilators, I’d like to examine the patient, and then I’ll need to get that scan… (R6, simulation 1)*

In his stimulated-recall session, R6 explained this comment as being useful both to N6 and to himself:

*I’m talking to the nurse there. I mean, I’m talking to myself, but I usually speak out loud because it helps me summarize the case, and this way nurses know what’s going on. (R6, stimulated-recall session 1)*

This shows the way in which nurses were often informed of residents’ reasoning, without being given the opportunity to be more active in the process.

#### Nurses: Stronger role perceptions, weaker actions

Nurses’ actions in simulations did not live up to their role perceptions for “Planning” (which, in our data, was specific to this profession, and refers to nurses’ organizing and prioritizing patient care), and for “Providing feedback and support”.

*Residents don’t always have a lot of experience, so in a way, we need to support them. Obviously, we won’t replace them, but I think that we can support them. (N6, interview)*

In simulations, residents often thanked nurses for their suggestions and acknowledged their contribution to patient care. Nurses, on the other hand, did not do so, despite enjoying working with residents:

*It’s amazing, R6 really is available. He’s available, our collaboration went well, he didn’t get nervous or stressed. (N6, stimulated-recall session 2)*

## Discussion

This exploration of internal medicine residents’ and nurses’ professional roles shows a modest relationship between role perceptions and actions. In this study, we 1) identified different components that residents and nurses do or do not consider as part of their roles, 2) assessed whether these components were translated into action using kappa statistics, and 3) qualitatively evaluated the strength and the nature of the relationship between role perceptions and actions. We identified nine role components common to both residents and nurses which reflect similarities in their activities, as shown in other studies [30]. Other role components were, however, more specific to either residents or to nurses. These components stressed the leadership role associated with residents [17, 31, 32], who were viewed as having more knowledge and deeper understanding of patients’ condition, and as being better able to train nurses on clinical matters. On the other hand, nursing was portrayed as a supporting role regarding patient management: nurses contributed to reasoning; they made suggestions, and verbally planned patient care around physicians’ decisions [17].

Given the importance of role clarity with regard to IPC [3, 4], we sought to delve deeper into this issue by analyzing whether or not various components of residents’ and nurses’ roles were translated into action. Role components for which participants’ actions matched their perceptions may be understood as favoring collaborative practice. We have shown that residents’ role perceptions aligned with their actions for “Contributing to team building”. Residents viewed trust and effective communication as important aspects of their role. They made sure to acknowledge nurses’ contribution to patient care and to listen to their suggestions in simulations. Nurses’ role perceptions strongly concurred with their actions on “Sharing technical information” with one another. For both residents and nurses, effective communication, be it to contribute to team dynamics or to share clinical information, was perceived as an important role component and was performed in simulations. As communication has been highlighted as being essential to IPC [4, 18, 19], concordance on this aspect is paramount. In fact, communication failure as a result of role misconceptions [33] may lead to the development or to the reinforcement of stereotypical views of professions [18, 34, 35]. Poor communication may also lead to tensions and conflicts within healthcare teams [36] and to regrets associated with the provision of care [37]. Concordance between role perceptions and actions for “Contributing to team building” and “Sharing technical information” therefore positively contributed to collaborative practice.

On the other hand, some components of participants’ roles lacked clarity, as we noted differences between residents’ and nurses’ role perceptions and their actions. This result was highlighted by the modest statistical relationship between role perceptions and actions, as well as by our qualitative analysis. Some role components were weakly expressed as being part of participants’ professional roles, but performed in simulations. For residents, this was the case for “Having common goals”. As it represents another key element of IPC [3], participants’ not mentioning it raises concerns. Recent research has demonstrated that, even though healthcare professionals share a common interest in improving patients’ health, team members often have different and competing priorities [38]. Lingard and colleagues used the terms of convergence and divergence to refer to situations where members of the healthcare team agreed or differed on patient care (e.g., identifying the main issues, deciding on a treatment plan, or evaluating how well a patient was). They concluded that convergence and divergence affected team competence in ways that could not be predetermined: while divergence between team members could generate tensions, it could also shed light on unclear areas that could then be discussed [38].

Nurses mentioned “Making suggestions” only weakly in interviews, but frequently performed it in simulations. This may reflect the ongoing “doctor-nurse game” [11, 12, 39]. While nurses may not be aware of it as a result of the traditional division of tasks between physicians and nurses [20, 40, 41], they often helped residents by making suggestions and taking initiative.

Differences between role perceptions and actions were also observed in role components for which participants performed what they had mentioned only weakly. Residents’ actions failed to live up to their views on “Depending on the other”. While they claimed that nurses should be given greater responsibility and recognition, residents’ actions in simulations and comments during stimulated-recall sessions did not align with their role perceptions. Similarly, nurses’ actions did not match their perceptions on “Providing feedback and support”, which they considered to be part of their role. These results may be indicative of some persistence of traditional patterns of interactions [20, 40, 41].

We have identified gaps between healthcare professionals’ role perceptions and their actions. As ambiguity with regard to professional roles may lead to conflicts within teams and may have negative effects on patient care [42], addressing these ambiguities is paramount. Despite recent efforts to promote IPC in healthcare professionals’ education, most educational programs are still profession-specific, socializing students into viewing professional roles in a traditional way [43]. However, recent research suggests that rather than being assigned, professional roles are fluid and vary depending on the context and on individuals [44]. Our results align with this idea. Residents and nurses mentioned different components of their professional roles. Some role components were similar for both professional groups, and some did not translate to simulations. Educators need to raise residents’ and nurses’ awareness of the gaps that may exist between their role perceptions and practice in the clinical setting, and to introduce them to the idea of more fluid boundaries between professional roles.

Limitations

Our study comes with limitations. While simulation scenarios had been designed to represent a range of non-urgent and urgent situations that are commonly encountered in general internal medicine, they may not have enabled participants to strongly perform all the role components they had mentioned in interviews. Field observations would have generated results that were more comprehensive in this regard, but they would not have permitted to standardize cases for all participants. The scenarios we developed may also balance the fact that our research was conducted in a single internal medicine ward. As we used typical internal medicine scenarios for simulations, the role components that we identified may be relevant to other internal medicine structures. Furthermore, as we wanted simulations to reflect actual practice, resident-nurse pairs were randomly created and were not based on matching role perceptions. Thus, our results may have been influenced by whether or not participants in each pair had similar role perceptions. A further limitation of our study is the fact that we used role components that had been identified in interviews and evaluated whether and how strongly they were performed in simulations. These therefore represent role components that participants viewed as being important, and do not represent an exhaustive list of all role components associated with a profession. To minimize the potential difference between the nature of data collected in interviews and in simulations, we took the following precautions: first, we contextualized interviews with the clinical vignettes. This allowed our participants to discuss their role perceptions both as ideals and as actions they would take on the ward. Second, as we used role components that had emerged from our interview data to assess those performed in simulations, participants themselves served as references. Finally, the stimulated-recall sessions that followed each simulation provided participants with the opportunity to comment on their actions and behaviors.

Implications for future research

Future areas of inquiry include extending our study to other institutions, so as to assess whether the same role components can be identified, as well as if similar discrepancies are observed between residents’ and nurses’ role perceptions and their actions. In addition, extending our research to other specialties, as well as to other health professions, may cast new light on our findings by emphasizing the influence of context. Specifically asking participants about identified gaps (which we were not able to do as stimulated-recall sessions immediately followed simulations) would provide rich information on healthcare professionals’ degree of awareness of these mismatches. A final implication of our study is related to the difference we have observed between role perceptions and actions. This difference emphasizes the need to evaluate students’ and professionals’ performances when assessing collaboration, rather than relying solely on self-reported data.

## Conclusions

Our study has identified a modest relationship between residents’ and nurses’ role perceptions and their actions, and examined this relationship by looking at different components of their professional roles. We identified areas in which residents and nurses experienced difficulties in translating components of their roles into action (e.g. “Having common goals”), which could in turn negatively affect IPC and patient care. These discrepant areas point to the need to address ambiguities associated with professional roles in interprofessional education. Focusing interprofessional training programs on these areas may represent a fruitful way to raise healthcare professionals’ awareness of the flexibility required when working in the clinical setting with regard to role boundaries. The discrepancies we noted between role perceptions and actions point to the importance of observing performance when seeking to evaluate interprofessional collaboration rather than relying on self-reported data alone.
